# The clinical characteristics and outcome of cryptococcal meningitis with AIDS in a tertiary hospital in China: an observational cohort study

**DOI:** 10.1186/s12879-020-05661-9

**Published:** 2020-12-01

**Authors:** Liang Wu, Jiang Xiao, Yangzi Song, Guiju Gao, Hongxin Zhao

**Affiliations:** 1 Clinical and Research Center of Infectious Diseases, Beijing Ditan Hospital, Capital Medical University, Beijing, China; 2 The National Clinical Key Department of Infectious Diseases, The Infectious Diseases Research Institute of Capital Medical University, The Infectious Diseases Center of Beijing Ditan Hospital, 8 Jingshundong Street, Chaoyang District, Beijing, 100015 China

**Keywords:** Cryptococcal meningitis, AIDS, Cerebral hernia, Consciousness disorder, Duration of induction therapy, Shunt decompression therapy

## Abstract

**Background:**

Despite the profound impact of antiretroviral therapy in the control of AIDS mortality, central nervous system opportunistic infections remains a significant burden in AIDS patients. This retrospective study aims to elucidate the clinical characteristics, outcome and risk factors of cryptococcal meningitis (CM) poor prognosis in AIDS patients from a tertiary hospital in China.

**Methods:**

Clinical data from 128 patients admitted in Beijing Ditan Hospital, Capital Medical University from November 2008 to November 2017 was collected. The cohort was stratified based on treatment outcome (effective 79%, and ineffective 21%), and Multivariate Logistic regression analysis used to identify risk factors of poor disease prognosis.

**Results:**

Age, incidence of cerebral infarction, the proportion of consciousness disorder, and fasting plasma glucose was higher in the ineffective treatment group than the effective treatment group. The duration of treatment in the induction period of the ineffective group was significantly shorter than that of the effective group. Multivariate Logistic regression analysis indicated that the occurrence of cerebral hernia and consciousness disorder were risk factors for the prognosis of AIDS patients with CM infection, while the duration of treatment in the induction period was a indicative of a better prognosis in AIDS with CM infection complications. Finally, shunt decompression therapy correlated with a better disease outcome.

**Conclusions:**

This retrospective study exposes the main risk factors associated with worse disease prognosis in AIDS patients with CM infection complications.

## Background

Nearly 30 years after the emergence of antiretroviral therapy (ART), the central nervous system (CNS) opportunistic infection (OI) is still the main cause of illness and death in patients with AIDS (Acquired Immunodeficiency Syndrome) [1]. Human Immunodeficiency Virus (HIV) -related central nervous system infections can be caused by a variety of microorganisms, including *Mycobacterium tuberculosis*, new cryptococcus, cytomegalovirus, and toxoplasma. Cryptococcal meningitis (CM) is one of the most common causes of AIDS-related deaths in the world [2], and it is also the most common fungal infection of the central nervous system, which is characterized by difficult treatment, high mortality and long course of disease. Clinically, the presence of a cerebral hernia, consciousness disorder, hydrocephalus, visual impairment, or intracranial pressure > 300mmH_2_O often indicates a poor prognosis in patients with CM [3]. Furthermore, increased intracranial pressure is positively correlated with CM morbidity and mortality in AIDS patients [4]. Initial intracranial pressure ≥ 250 mmH_2_O was found in 65% of CM patients, and consciousness disorder was an independent risk factor for death in CM patients [5]. Similarly, treatment with amphotericin B (AmB) for less than 14 days was associated with a higher 90-day mortality in CM patients [6]. Early internal drainage is the key factor for better prognosis, and cerebrospinal fluid (CSF) positive for cryptococcus before surgery is not a contraindication [3].

Despite well described in western countries, the clinical characteristics and outcome of CM infection in AIDS patients remains less clear in China. In this study, clinical data of 128 AIDS patients with CM infection complications, admitted to the Beijing Ditan hospital in China for the past 10 years, were collected, and prognostic factors were analyzed to guide early clinical identification of patients with severe CM, timely rescue measures and improved prognosis.

## Methods

### Subjects

We collected data from 128 AIDS patients with CM infection, hospitalized in Beijing Ditan hospital from November 2008 to November 2017. A retrospective study was performed in an observation cohort, approved by institutional review board (IRB) of Beijing Ditan Hospital, the Capital Medical University.

### Criteria of diagnosis and treatment

The diagnosis and treatment of Acquired Immunodeficiency Syndrome (AIDS) referred to *the Guidelines on HIV/AIDS Diagnosis and Treatment in China (2018 edition)* [7]. All the patients were confirmed to be positive for anti-HIV-1 antibody by Western blot test (WB). ART was initiated 4-weeks after receiving Amphotericin B (AmB) or 6-weeks after receiving fluconazole (FLU) in AIDS patients with CM infection.

The diagnosis of CM followed *the Expert Consensus on the Diagnosis and Treatment of Cryptococcal Meningitis* [8], and based on CSF cryptococcal antigens, smear ink staining and fungal culture. The treatment of CM followed the expert consensus, and standard treatment regimens included AmB ± 5-fluorocytosine (5FC) and FLU±5FC.

### Data collection and definitions

Demographic data included gender and age, while collected clinical data included symptoms, signs, complications and treatment regimens of CM. Laboratory data included routine test, biochemistry and pathogenic detection of cerebrospinal fluids, blood routine tests, electrolytes, C-reactive protein, procalcitonin, fasting plasma glucose, CD4 cell counts and HIV viral load.

Clinical efficacy of anti-CM treatment was evaluated as: ① Complete response: meningitis symptoms and signs disappeared, CSF routine and biochemical examination returned to normal, ink staining and fungal culture turned negative; ② Partial response: meningitis symptoms and signs improved, CSF routine and biochemical examination improved, ink staining and/or fungal culture turned negative; ③ Invalid: no improvement in symptoms and signs of meningitis, no improvement in routine and biochemical examination of CSF, still positive in ink staining and/or fungal culture; ④ Death.

Based on the clinical efficacy criteria, patients were stratified in effective (Complete or partial response, ① + ②) and ineffective (invalid or death ③ + ④) groups, and compared for age, symptoms, signs, complications, CSF examination, blood examination, treatment timing, treatment plan and course of treatment during induction period. The end point of observation was patient discharge.

### Statistical analysis

SPSS 22.0 software was used for data analysis. The measurement data conforming to the normal distribution were expressed as (X ± S), and t-test was used for comparison between groups. Non-normal distribution data were statistically described by median and quartile, and non-parametric rank sum test was used for comparison between groups. The enumeration data were expressed as examples or percentages and X^2^ test was used for comparison between groups. Logistic regression analysis was performed to evaluate risk factors of prognosis among AIDS patients with CM. For all analysis, a *p* value < 0.05 was considered statistically significant.

## Results

### The clinical characteristics and outcomes

**Fig. 1:**
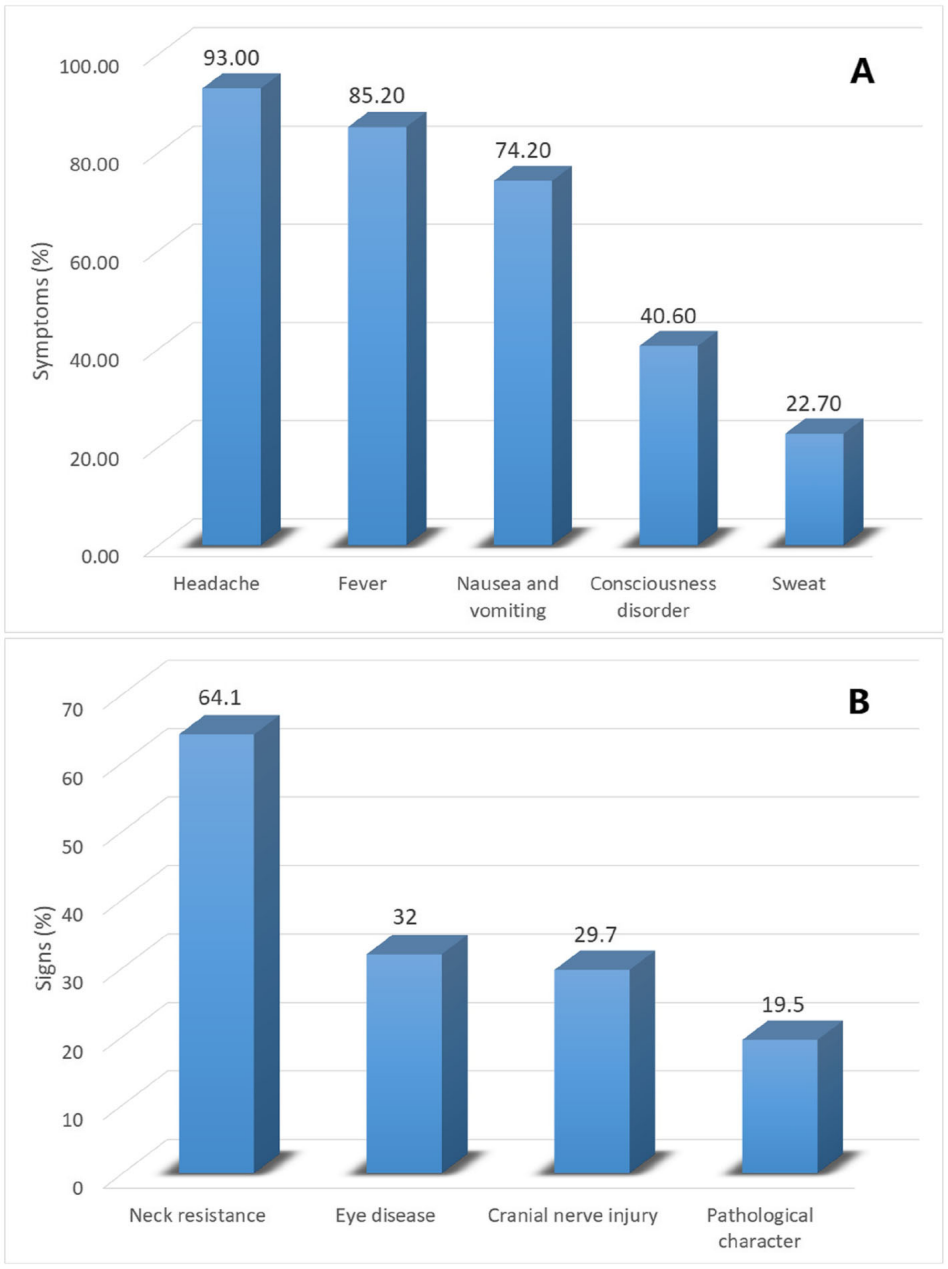
Occurrence rate of symptoms and signs of AIDS patient with CM

A total of 128 cases diagnosed AIDS patients with CM were retrospectively studied, including 113 males and 15 females, aged 14–74 years. The clinical characteristics of these patients are presented in detail in Fig. 1 and Table 1.

**Table 1:** Clinical characteristics and data of AIDS patient with CM in the study cohort

Variables	Total(*n* = 128)	Effective cohort(*n* = 101)	Ineffective cohort(*n* = 27)	Statistic	*P-*value
age	37.5 (30.25, 46.75)	36 (30, 44.5)	42.3 ± 13.5	−1.981	0.048
Sex (cases)					
Male	113 (88.3%)	92 (91.1%)	21 (77.8%)	−1.903	0.057
Female	15 (11.7)	9 (8.9%)	6 (22.2%)		
Complications (cases)					
Tuberculous meningitis	14 (10.9%)	9 (8.9)	5 (18.5%)	−1.415	0.157
Purulent meningitis	9 (7%)	6 (5.9%)	3 (11.1%)	−0.930	0.352
Cryptococcal pneumonia	31 (24.2%)	26 (25.7%)	5 (18.5%)	−0.775	0.438
Epilepsy	11 (8.6%)	10 (9.9%)	1 (3.7%)	−1.017	0.309
Optic papillary edema	6 (4.7%)	5 (5%)	1 (3.7%)	−0.271	0.786
Deafness	6 (4.7)	6 (5.9%)	0 (0%)	−1.292	0.196
Hydrocephalus	3 (2.3%)	2 (2%)	1 (3.7%)	−0.524	0.600
Cerebral hernia	4 (3.1%)	2 (2%)	2 (7.4%)	−1.434	0.152
Cerebral infarction	8 (6.3%)	4 (4%)	4 (14.8%)	−2.062	0.039
Signs and symptoms (cases)					
Fever	109 (85.2%)	89 (88.1%)	20 (74.1%)	−1.816	0.069
Sweat	29 (22.7%)	24 (23.8%)	5 (18.5%)	−0.576	0.565
Headache	119 (93%)	93 (92.1%)	26 (96.3%)	−0.758	0.448
Nausea and vomiting	95 (74.2%)	76 (75.2%)	19 (70.4%)	−0.513	0.608
Consciousness disorder	52 (40.6%)	36 (35.6%)	16 (59.3%)	−2.211	0.027
Eye disease	41 (32%)	31 (30.7%)	10 (37%)	−0.625	0.532
Cranial nerve injury	38 (29.7%)	27 (26.7%)	11 (40.7%)	−1.410	0.159
Neck resistance	82 (64.1%)	65 (64.4%)	17 (63%)	−0.134	0.894
Pathological signs	25 (19.5%)	19 (18.8)	6 (22.2%)	−0.396	0.692
CSF pressure (mmH_2_O)					
< 180	25 (19.5%)	23 (22.8%)	2 (7.4%)	−1.741	0.082
180–250	38 (29.7%)	30 (29.7%)	8 (29.6%)		
> 250	65 (50.8%)	48 (47.5%)	17 (63%)		
CSF routine and biochemical					
WBC (cells/μl)	20 (10, 59)	20 (10, 61.5)	23 (10, 50)	−0.138	0.891
TP(g/L)	40.4 (30.8, 73.85)	38.6 (30.5, 64.9)	50.3 (30.9, 91)	−1.256	0.209
GLU (mmol/L)	2.45 ± 0.95	2.51 ± 0.89	2.24 ± 1.15	1.110	0.275
CL (mmol/L)	117.9 ± 6.38	118.47 ± 5.78	115.76 ± 8.02	1.647	0.109
Etiological examination positive (cases)					
CSF Ink stain	105 (82%)	84 (83.2%)	21 (77.8%)	−0.646	0.519
CSF Cryptococcus antigen	128 (100%)	101 (100%)	27 (100%)	0.000	1.000
CSF Cryptococcus culture	75 (58.6%)	59 (58.4%)	16 (59.3%)	−0.079	0.937
Blood Cryptococcus antigen	125 (97.7%)	99 (98%)	26 (96.3%)	−0.524	0.600
Blood Cryptococcus culture	67 (52.3%)	54 (53.5%)	13 (48.1%)	−0.489	0.625
WBC(×10^9^/L)	4.97 (3.37, 6.59)	4.94 (3.19, 6.82)	5.57 ± 2.04	−0.873	0.383
NE%(%)	78.26 (66.64, 83.26)	78.11 (65.8, 82.58)	76.63 ± 13.56	−1.171	0.242
LY%(%)	12.97 (7.92, 20.15)	12.92 (7.97, 20.85)	14.89 ± 11.1	−0.447	0.655
HGB(g/L)	120.04 ± 22.59	118.76 ± 22.47	124.80 ± 22.84	−1.237	0.218
PLT(×10^9^/L)	175.5 (128.5, 226.25)	176 (117.5, 225.5)	166.50 ± 68.80	−0.517	0.605
ESR (mm/h)	41 (21.5, 66)	46.17 ± 27.77	27 (13, 68)	−1.481	0.139
CRP (mg/L)	13.05 (4.58, 38.88)	14.7 (5.46, 47.6)	7.5 (3.6, 31)	−1.273	0.203
PCT (ng/ml)	0.05 (0.05, 0.19)	0.06 (0.05, 0.21)	0.05 (0.05, 0.16)	−0.489	0.625
K (mmol/L)	3.58 ± 0.53	3.62 ± 0.53	3.43 ± 0.55	1.613	0.109
Na (mmol/L)	131.98 ± 5.51	132.09 ± 5.28	131.59 ± 6.38	0.417	0.677
Cl (mmol/L)	97.23 ± 5.54	97.42 ± 5.26	96.51 ± 6.54	0.756	0.451
GLU (mmol/L)	6.33 (5.67, 7.46)	6.11 (5.54, 7.04)	6.91 (6.23, 8.1)	−2.695	0.007
CD4(cells/μl)	22.5 (9.25, 47)	21 (9, 46)	26 (10, 55)	−0.973	0.331
HIV RNA (copies/ml)	68,466.5 (18,675.5, 186,018.5)	84,218 (14,286, 182,126)	31,659 (19,412, 190,202)	−1.072	0.284
Start/Unstart ART (cases)	32/96	25/76	7/20	0.016	0.901
Treatment timing/Course (weeks)	4 (3, 8)	4 (2, 8)	4 (3, 9)	−0.672	0.502
Therapeutic schedule (cases)					
AmB ± 5FC	8 (6.3%)	7 (6.9%)	1 (3.7%)	−1.825	0.068
FLU±5FC	57 (44.5%)	47 (46.5%)	10 (37%)		
Voriconazole	4 (3.1%)	4 (4.0%)	0 (0%)		
Mixed regimens	39 (30.5%)	31 (30.7%)	8 (29.6%)		
Non-standard treatment	20 (15.6%)	12 (11.9%)	8 (29.6%)		
Induction course (weeks)	5 (3, 10)	7 (4, 10)	2 (1, 5)	−4.398	< 0.001

**Note:** *CSF* Cerebrospinal fluid, *WBC* White blood cell, *TP* Total protein, *GLU* Glucose, *CL* Chloride ion, *NE%* Neutrophil%, *LY%* Lymphocyte, *HGB* Hemoglobin, *PLT* Platelet, *ESR* Erythrocyte sedimentation rate, *CRP* C reactive protein, *PCT* Procalcitonin, *K* Potassium ion, *Na* Sodium ion, *CD4* CD4+ T lymphocyte count, *ART* Antiretroviral therapy, *AmB ± 5FC* Amphotericin B ± 5-fluorocytosine, *FLU±5FC* Fluconazole±5-fluorocytosine, *Median (Q1, Q3)* Median based on 25th and 75th percentiles

The course of CM at admission ranged from 1 to 156 weeks, with a median of 4 weeks (3, 8 weeks). Hospitalization time ranged from 1 to 210 days, with a median of 20 days (7, 41 days).

**Fig. 2:**
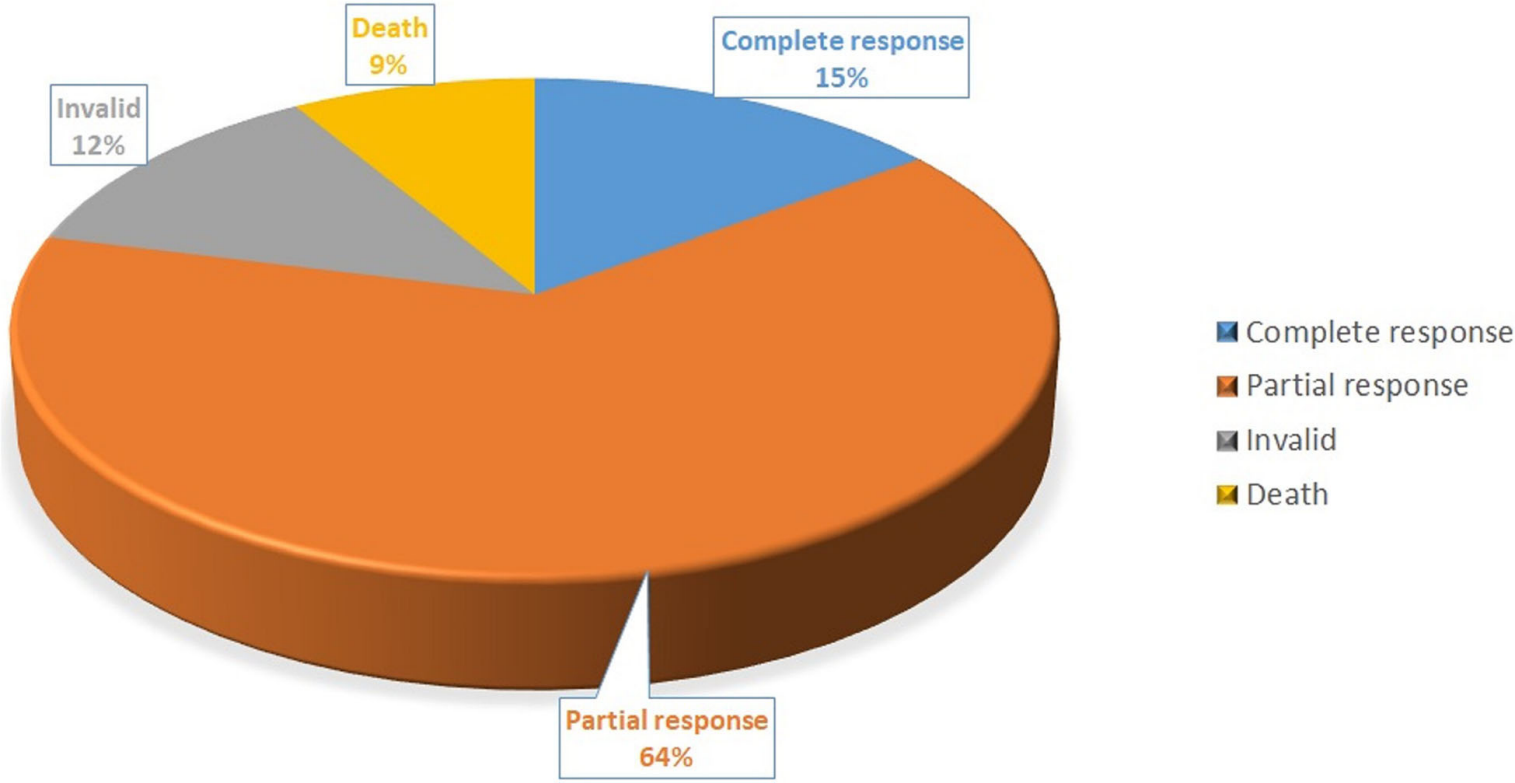
Schematic diagram of outcome of AIDS patient with CM in the study cohort

Patients receiving anti-CM treatment, presented different clinical outcomes; with 15% of cases showing a complete response, 64% a partial response, 12% invalid and 9% death (Fig. 2). After treatment, clinical symptoms disappeared in 25.8% of cases and improved in 60.2%. Routine test and biochemistry of CSF returned to normal or improved in 34.4 and 35.2% of cases, respectively, while ink staining in CSF returned to negativity in 35.9% of patients (Table 2).

**Table 2:** Number and time of transition of AIDS patient with CM in the study cohort

Outcome	Number of cases (persons)	Time (weeks)
**Symptoms**		
Disappeared	33 (25.8%)	2 (1.5, 3)
Improved	77 (60.2%)	3 (2, 4)
Not improved	18 (14%)	–
**CSF**		
Return to Normal	44 (34.4%)	1 (1, 2.75)
Improved	45 (35.2%)	2 (2, 4)
No Improved	25 (19.5%)	–
No rechecked	14 (10.9%)	–
**CSF ink stains turn to negative**	46 (35.9%)	16 (7, 30)

**Note**: *CSF* Cerebrospinal fluid, *Median (Q1, Q3)* Median based on 25th and 75th percentiles

### Comparison of clinical characteristics between effective and ineffective cohorts receiving anti-CM treatment

The age of the ineffective group (invalid or death) was higher than that of the effective group (complete or partial response, *p* = 0.048). Additionally, the incidence of cerebral infarction in the group with ineffective clinical treatment was higher than that in the group with effective clinical treatment (*p* = 0.039). Based on symptom and sign factors, the proportion of consciousness disorder was higher in the ineffective clinical treatment group (*p* = 0.027). There were no significant differences in CSF pressure, CSF routine and biochemistry, CSF ink staining, CSF and blood cryptococcus antigen and cryptococcus culture between the two groups. Furthermore, fasting plasma glucose was the only biochemical parameter significantly increased in ineffective treatment group (*p* = 0.007). There were no significant differences in blood routine, infection markers, electrolytes, CD4 count and HIVRNA load between the two groups. In terms of treatment timing/course and treatment regimen, there was no statistical difference between the effective and ineffective group in the clinical treatment. However, the duration of treatment in the induction period of the ineffective group was significantly shorter (*p* < 0.001) (Table 1).

### The risk factors of prognosis among patients with CM

Multivariate Logistic regression analysis indicated that the occurrence of cerebral hernia (OR 404.617, 95%CI 6.052, 27,050.598, *p* = 0.005) and consciousness disorder (OR 4.376, 95%CI 1.539, 12.444, *p* = 0.006) were risk factors for the prognosis of AIDS patients with CM. The occurrence of cerebral hernia increased the risk of poor prognosis in AIDS patients with CM infection by 405 times, and the occurrence of consciousness disorder increased the risk of poor prognosis by 4.4 times. Contrastingly, the duration of treatment in the induction period (OR 0.683, 95%CI 0.560, 0.834, *p* < 0.001) was a protective factor associated with better disease prognosis in AIDS patients with CM infection complications. For each week of treatment in the induction period, the risk of poor prognosis was reduced by 0.683 times, and the effective outcome of clinical treatment was increased by 1.5 times (Table 3).

**Table 3:** Logistic regression analysis of prognostic factors in AIDS patients with CM

			*P-*value	*OR*	95% CI of OR	
	Cerebral hernia		0.005	404.617	6.052	27,050.598
	Consciousness disorder		0.006	4.376	1.539	12.444
	Induction course		< 0.001	0.683	0.560	0.834
	Constant		< 0.001	< 0.001		

**Note**: *CI* Confidence Interval

### Better disease outcome in CM patients receiving shunt decompression therapy

**Fig. 3:**
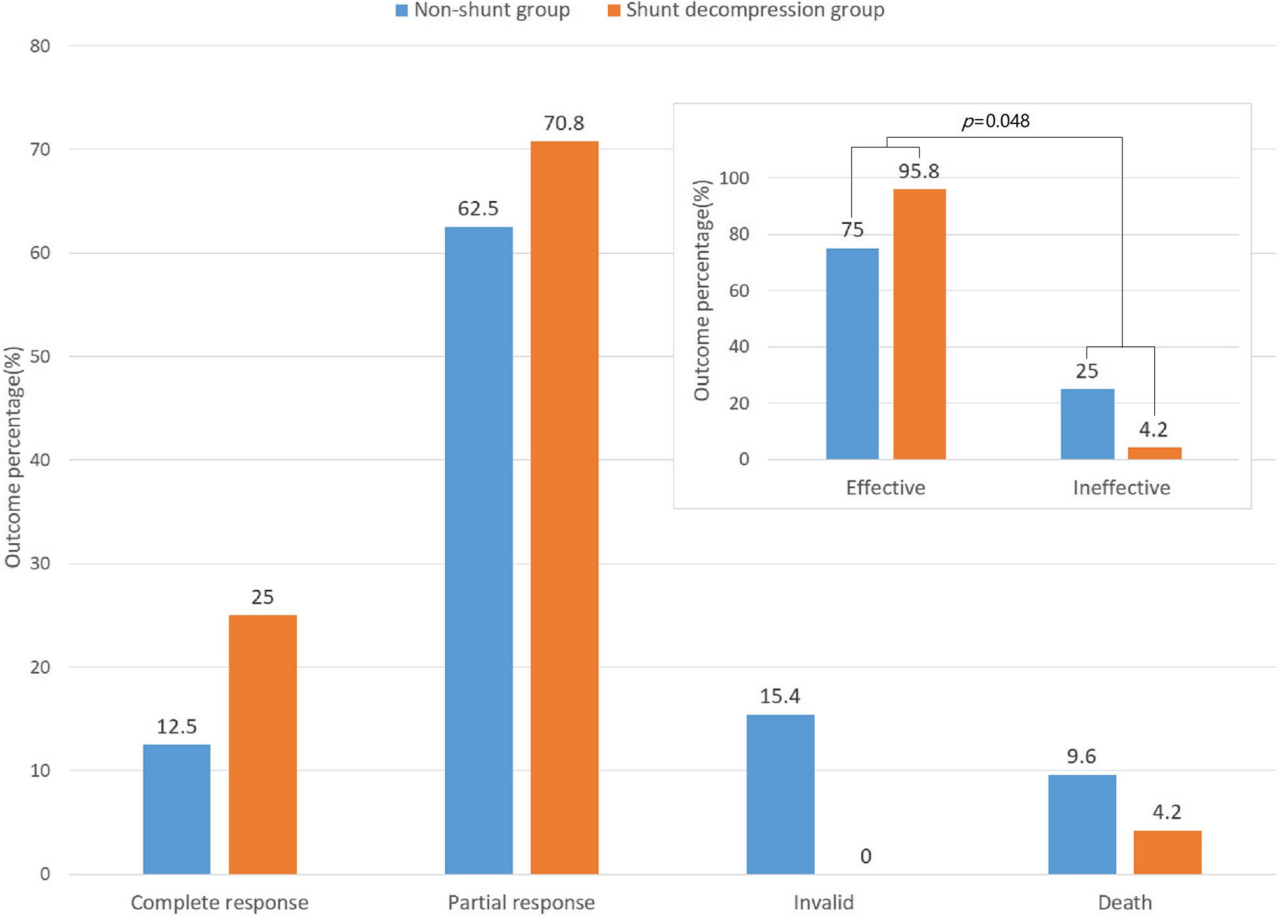
Outcome of AIDS patient with CM treated with shunt decompression/non-shunt therapy

In this study, besides anti-CM treatment, 24 cases of shunt decompression treatment were performed, including 5 cases of ventriculoperitoneal shunts (VP) (including 1 case undergoing lumboperitoneal shunts again due to catheter blockage) and 19 cases of lumboperitoneal shunts (LP) (including 1 case undergoing extubation and recatheterization due to infection, and 1 case with extubation due to catheter blockage). Median duration from CM diagnosis to shunt decompression was 7 weeks (5, 10 weeks). In patients receiving shunt decompression therapy, 6 and 17 showed complete or partial response, respectively, while 1 succumbed to disease, translating to a 95.8% clinical response rate. Among patients not receiving shunt decompression therapy, complete and partial response occurred in 13 and 65 cases, respectively, with 16 invalid cases, and 10 deaths, reflecting a 75% clinical response rate. Continuity correction Chi-Square test indicated a significant statistical difference between CM patients receiving or not receiving shunt decompression therapy (*p* = 0.048). Overall, patients receiving shunt decompression treatment had a better disease outcome (Fig. 3).

## Discussion

The mortality of AIDS patients with CM in different countries ranges from 10 to 43% [9]. It has been reported that the 2-weeks mortality rate of AIDS patients with CM is 17%, and 10-weeks mortality rate can reach 34%, and the overall 1-year mortality rate is 41% [10]. Even in developed countries, with the best antifungal and ART regimen, the mortality of HIV-associated CM within 10 weeks is still 10 to 25% [11]. Increased intracranial pressure is positively correlated with CM incidence and mortality of CM, and is positively correlated with the increase of cerebrospinal fluid fungal load [4]. Early effective control of intracranial hypertension is one of the most critical determining factors for the outcome of CM patients, and for successful antifungal therapy with reduced mortality [12]. Comprehensive treatment including anti-cryptococcus agents, intracranial pressure management and ART are the key to reduce the mortality of AIDS patients with CM infections complications [13, 14].

In this study, we found that 9% mortality rate among AIDS patients with CM receiving anti-CM treatment, which is in agreement with previous literature reports worldwide. The anti-cryptococcus treatment in induction period of the patients mainly included FLU ±5-FC (44.5%), sequential FLU ±5-FC, AmB ± 5-FC (30.5%), and AmB ± 5-FC (6.3%). Most of the patients chose FLU (800-1200 mg / d) ± 5-FC (100 mg / kg.d), due to pronounced side effects of AmB and the price of liposomal AmB. It was reported that the prognosis in AIDS patients with CM receiving AmB ± 5-FC regimen may be better than other regimens, and that anti-CM regimen, FLU ±5- FC, may be an alternative for patients intolerant AmB [15]. A 10-year data-control study indicated that Chinese AIDS patients with CM receiving anti-CM regimens, FLU ±5- FC and AmB ± 5-FC, presented no statistically significant difference in CSF pressure, CSF cryptococcal number, CSF protein, CSF WBC and disease outcome [16]. Molloy et al. reported that the induction period treatment regimens of 2-weeks FLU (1200 mg/d) + 5FC (100 mg/kg.d), 1-week AmB (1 mg/kg.d) and 2-weeks AmB (1 mg/kg.d) had similar mortality rates at 2 weeks and 10 weeks for AIDS patients with CM infection [17]. In accordance to the guideline of diagnosis and treatment of cryptococcal infection of US CDC, WHO and China [8, 12, 18], whatever not less than 2 weeks, or 4 weeks, or even 10–12 weeks, induction treatment should be ended when CSF cryptococcus culture turn negative, followed by consolidation or maintenance treatment. Our study indicated that the duration of induction period treatment in the ineffective group was significantly shorter than that in the effective group (*p* < 0.001), highlighting the importance of adequate duration of induction period treatment in AIDS patients with CM receiving anti-CM treatment.

We also found that the age and fasting plasma glucose of the clinically ineffective group were higher, and the incidence of cerebral infarction and consciousness disorder was higher than that of the effective group. A 10-weeks follow-up study in Thailand, Uganda, South Africa and other countries indicated that age over 50, changes in mental state, high fungal burden of CSF, removal rate of cryptococcus, and peripheral blood WBC > 10 × 10^9^/L were independently correlated with early mortality of patients with AIDS complicated with CM infection [10]. The mechanism of cerebral infarction caused by CM may be related to extensive fibrosis of the subarachnoid space caused by cryptococcus, mechanically compression of the small veins, and increased blood flow resistance [19]. However, the correlation between the level of fasting plasma glucose, the occurrence of cerebral infarction and the poor prognosis of AIDS patients with CM infection complications has not been previously reported.

The increase of intracranial pressure is positively correlated with the morbidity and mortality of CM [4]. Increased intracranial pressure is a prerequisite for the formation of cerebral hernia, which occurs when intracranial pressure is unevenly distributed. Cerebral hernia itself has a very poor prognosis, high mortality and disability rate. Consciousness disorder is an independent risk factor for death in CM patients [5]. Lofgren et al. proposed that changes in mental state of CM patients lead to poor prognosis, and the change in mental state may be more related to the host immune response than the cryptococcus burden [20]. In this study, we found that occurrence of cerebral hernia and consciousness disorder are risk factors for the poor prognosis of AIDS patients with CM, which was consistent with conclusions reported in previous literature.

In this study, we found that the duration of treatment in the induction period is a protective factor for better prognosis of AIDS patients with CM, which is rarely reported at home and abroad. Considering the difficulty of CM treatment, the high fatality rate, the long course of disease, and that CM clearance rate in CSF is significantly related to early-stage mortality; and that the end of the induction period treatment is based on the CSF cryptococcus culture negative conversion; our study suggests that efficient anticryptococcal therapy for a sufficient induction period is especially critical to improve disease prognosis.

Another important finding of this study is that the prognosis of patients receiving shunt decompression treatment is better than that of patients without shunt treatment. Despite severe immunodeficiency and persistent CNS cryptococcus infection, AIDS and CM patients with increased intracranial pressure have indication for LP and/ or VP shunts [21]. Shunt surgery is usually a sustainable way to relieve the symptoms associated with increased intracranial pressure [22]. Baddley et al. [23] reported that shunt surgery may improve the prognosis associated with increased intracranial pressure in CM, which was consistent with our results.

This study is a clinical retrospective study. Due to limited access of follow-up data from participating patients, the impact of the clinical factors here analyzed on long-term prognosis was unattainable, and the collected ART data could not be evaluated. Additionally, the personalized choice of treatment options during the induction period lead to a biased distribution of treatment options, which limited the reliability of the conclusions obtained in terms of treatment timing/course of disease and treatment options. Future studies should address this limitations with more extensive and balanced cohorts.

## Conclusions

Cerebral hernia and consciousness disorder are risk factors for prognosis of AIDS patients with CM infection; and the duration of induction therapy is a protective factor for disease prognosis. Efficient anti-cryptococcal therapy for sufficient induction period is especially critical for improving prognosis, and shunt decompression therapy may improve the prognosis of AIDS patients with CM infection complications. All these results will guide early identification of severe patients and early rescue measure, to improve the prognosis and reduce mortality rates.

## Data Availability

The data used to support the findings of this study are available from the corresponding author upon request.

## Abbreviations

5-FC: 5-fluorocytosine
AIDS: Acquired Immunodeficiency Syndrome
AmB: Amphotericin B
ART: Antiretroviral therapy
CM: Cryptococcal meningitis
CNS: Central nervous system
CSF: Cerebrospinal fluid
FLU: Fluconazole
HIV: Human Immunodeficiency Virus
LP: Lumboperitoneal shunts
OI: Opportunistic infection
VP: Ventriculoperitoneal shunts
WB: Western blot test
